# Effects of *Rhodotorula* Yeast Culture on Nutrient Apparent Digestibility and Rumen Health in Sheep

**DOI:** 10.3390/biology15050390

**Published:** 2026-02-27

**Authors:** Jie Ma, Jianlong Dang, Huiru Ma, Guang Yang, Ke Wang, Xinyu Lu, Xiangtan Su, Xinhao Zhang, Feilong Liu, Aiqin Gao

**Affiliations:** 1 College of Animal Science, Inner Mongolia Agricultural University, Hohhot 010018, China; 18195167236@163.com (J.M.);; 2 Department of Agronomy, Hetao College, Bayannur 015000, China

**Keywords:** *Rhodotorula* yeast culture, sheep, rumen, fungal microbiota

## Abstract

The potential of *Rhodotorula* yeast culture (RYC) in animal production remains underexplored. This study investigated the effects of RYC supplementation on nutrient apparent digestibility, rumen tissue morphology, fermentation parameters, and fungal microbiota in sheep. Twenty-four three-month-old male Dorper × Han crossbred sheep (weight 36 ± 4 kg) were selected and randomly divided into four groups, with six sheep in each group: the control group (CON) was fed a basal diet, and the three treatment groups were supplemented with 10, 20, or 40 g/d of RYC (RYC10, RYC20, RYC40), respectively. The results showed that RYC supplementation significantly increased (*p* < 0.05) the apparent digestibility of dry matter, crude protein, neutral detergent fiber, and acid detergent fiber, and the apparent digestibility of CP and ADF was significantly higher in the RYC20 than in the other groups (*p* < 0.05). Rumen papillae length and muscular layer thickness were significantly greater (*p* < 0.05) in RYC-treated groups compared to the CON group, and the RYC20 group exhibited significantly greater rumen papilla length and muscularis propria thickness than the other experimental groups (*p* < 0.05). Furthermore, ruminal pH and bacterial crude protein content were significantly elevated (*p* < 0.05), while ammonia nitrogen concentration was significantly reduced (*p* < 0.05). The RYC40 group exhibited significantly higher rumen pH and BCP concentrations, and significantly lower NH3-N concentration, compared to the other experimental groups (*p* < 0.05). The concentrations of acetate, propionate, butyrate, and total volatile fatty acids were also significantly higher (*p* < 0.05) in RYC groups. For RYC20, rumen acetic acid, propionic acid, butyric acid, isobutyric acid, total volatile fatty acid content and the acetate-to-propionate ratio were significantly higher than those of the other experimental groups (*p* < 0.05). Analysis of fungal community revealed that RYC increased the relative abundance of fibrolytic fungi (e.g., *Neocallimastix*, *Caecomyce, Piromyces*). Supplementation of RYC at 20 g/d optimizes apparent nutrient digestibility and rumen tissue development in ruminants, while maintaining favorable rumen fermentation characteristics and selectively enhancing the growth of core fibrolytic fungi; this dosage achieves the optimal balance of biological performance and economic feasibility, and is thus recommended as the optimal practical supplementation dosage for ruminant production.

## Simple Summary

The use of feed additives to ensure sheep health has attracted increasing attention under China’s current policy of completely prohibiting the addition of antibiotics to feed. As a novel strain in the yeast family, *Rhodotorula* yeast corpora is rich in nutrients such as proteins, hepatic sugars, advanced unsaturated fatty acids, carotenoids and natural growth hormones. However, the application of *Rhodotorula* yeast culture (RYC) as a novel feed additive in ruminant production has not been widely studied. Therefore, this experiment was conducted to investigate the effects of adding different levels of RYC to diets on nutrient apparent digestibility, rumen tissue morphology, rumen fermentation parameters and rumen fungal flora of sheep, to provide theoretical and practical bases for its application in sheep production. In summary, the addition of RYC to diets can contribute to maintaining a healthy sheep herd, increase the apparent digestibility of feed nutrients in sheep, improve the performance of sheep, promote the development of rumen tissues and improve rumen fermentation. At the same time, it is beneficial to enhance cellulolytic bacterial growth in the rumen, improve the utilization rate of fibrous material in sheep, and maintain rumen health. Supplementation of RYC at 20 g/d optimizes apparent nutrient digestibility and rumen tissue development in ruminants, while maintaining favorable rumen fermentation characteristics and selectively enhancing the growth of core fibrolytic fungi; this dosage achieves the optimal balance of biological performance and economic feasibility, and is thus recommended as the optimal practical supplementation dosage for ruminant production.

## 1. Introduction

The rumen, a unique digestive organ in ruminants, harbors diverse microorganisms including fungi, bacteria and protozoa [1], its function critically influences both ruminant physiology and production performance [2]. A symbiotic relationship prevails between the host and the rumen microbiota, whereby microbes supply essential nutrients crucial to ruminant development [3]. Dietary composition exerts the primary influence on the microbial community structure within the ruminant digestive tract [4]. However, intensive farming practices increasingly utilize high-concentrate diets, which elevate the risk of metabolic disorders, such as rumen acidosis, thereby compromising rumen health, threatening industry sustainability, and causing substantial production losses [5].

Driven by the global prohibition of antibiotic growth promoters (AGPs), research on eco-friendly feed additives—such as plant extracts and probiotics—has become a central focus in ruminant nutrition [6]. Following China’s full implementation of this ban, extensive studies on these alternatives have been initiated [7]. Yeast culture (YC) is a green additive probiotic, rich in yeast polysaccharides, mannan-oligosaccharides, vitamins, peptides and organic acids, which can be used as fermentation substrates and growth factors to promote the growth of beneficial bacteria in the gastrointestinal tract of ruminants [8]. YC polysaccharides modulate rumen microbiota growth, enhancing nutrient apparent digestibility and improving ruminant-derived product quality [9]. The probiotics in YC regulate the rumen microbial community structure, reduce lactic acid accumulation, and maintain ruminal pH homeostasis [10]. Extensive studies demonstrate that YC consequently enhances rumen pH, crude fiber digestibility, and growth performance [11], enhances overall immunity [12], and increases the structure and abundance of the microbiome [13]. Bacteria are the most abundant and diverse members in the rumen, with 10^10^ to 10^11^ cells per gram, and represent the most extensively studied group within the rumen ecosystem [14]. In contrast to bacteria, fungi are far less numerous, comprising 10^3^ to 10^5^ cells per gram, yet fungi have higher fibrolytic enzyme activities. Due to the extensive production of cellulase and xylanase, fungi serve as highly efficient fiber degraders and play a crucial role in the initial colonization and physical disruption of feed particles [15,16,17]. For a long time, anaerobic fungi have been considered to play a crucial role in fiber degradation within the rumen and are recognized as one of the primary colonizers of lignocellulosic substrates in ruminant diets [18]. Since fungal hyphae can penetrate and physically disrupt feed particles, more surface area is exposed for microbial colonization, thereby enhancing the degradation of plant biomass [19]. Research efforts aimed at linking the rumen microbiome to host efficiency have predominantly centered on bacterial communities. Given the functionally significant role fungi play in digestion, their contribution to ruminal fermentation has become a subject of increasing research interest [20]. Depletion of anaerobic fungal populations results in a significant reduction in the in vivo digestibility of feed in sheep [21]. Dietary supplementation of rumen-sourced fungal isolates improves feed digestion and promotes body weight gain in buffalo calves [22]. Paul et al. [23] demonstrated that anaerobic fungi, isolated from wild ruminant feces, possess potent lignocellulolytic activity and can enhance nutrient availability upon inoculation into other ruminants. However, to date, no studies have directly linked the native rumen fungal community to rumen health parameters in meat-producing sheep. Therefore, this study focused specifically on the fungal community.

*Rhodotorula* species, including *R. mucilaginosa* and *R. minuta,* are widely distributed fungi known for producing carotenoids, digestive enzymes, β-glucan, vitamins, and other active metabolites [24,25]. And it exhibits chemoorganoheterotrophic, thermotolerant, acidotolerant, and facultatively anaerobic characteristics [26,27]. Hu’s research indicated that *R. mucilaginosa* improved growth performance, enhanced antioxidant capacity, strengthened gastrointestinal digestion, and maintained the intestinal microbiological balance of piglets [25]. Sun et al. found that *R. mucilaginosa* improved the yolk color and increased the carotenoid content, thereby improving the intestinal health of hens [28]. Li et al. [29] incorporated the solid-state fermentation product of *R. mucilaginosa* into the diet of laying hens, which improved the duodenal morphology and enhanced the abundance and diversity of the gut microbiota. Chen et al. [30] found that dietary hydrolyzed yeast *R. mucilaginosa* improved the growth performance and antioxidant capacity of Nile Tilapia.

Existing research indicates that YC exerts certain effects on the growth performance, immune function and rumen health of sheep. And *R. mucilaginosa* is characterized by its ability to produce carotenoids. The main active component of RYC is carotenoids, among which β-carotene, as a precursor of vitamin A, not only improves animal growth performance and enhances immune function but also boosts antioxidant capacity and promotes intestinal development in animals. Therefore, the carotenoids in RYC exhibit potent antioxidant activity that mitigates oxidative stress in rumen microbes, thereby enhancing the activity of fibrolytic fungi—this provides direct evidence for the specific effects of carotenoids [31].

Therefore, we hypothesize that the *R. mucilaginosa* yeast culture (RYC) not only exhibits the biological activities typical of YC but also exerts the specific effects associated with carotenoids. Nevertheless, there are no studies exploring the effects of RYC on fattening sheep. Therefore, this experiment aimed to investigate the effects of adding different levels of RYC to diets on nutrient apparent digestibility, rumen tissue morphology, rumen fermentation parameters and rumen fungal flora of sheep, so as to explore whether RYC can be an alternative to antibiotic feed additives.

## 2. Materials and Methods

All animal experiments were conducted in accordance with protocols approved by the Animal Welfare and Ethics Committee of Inner Mongolia Agricultural University (approval number NND2022110).

### 2.1. Animals and Experimental Design

Twenty-four three-month-old male Dorper × Han crossbred sheep (weight 36 ± 4 kg) were selected and randomly divided into 4 groups, with 6 sheep in each group. This experiment was conducted at Inner Mongolia Fuchuan Breeding Technology Co., Ltd. (Bayannur, China). in a completely randomized experimental design. The experiment lasted 90 days, comprising a 15-day adaptation phase and a 75-day trial phase. The control group (CON) was fed a basal diet, while the experimental group was supplemented with 10 g (RYC10), 20 g (RYC20), and 40 g (RYC40) of *Rhodotorula* yeast culture per day to the basal diet, respectively. The formula and nutritional levels of the basal diets are shown in Table 1, formulated by Inner Mongolia Fuchuan Breeding Science and Technology Co. RYC (β-glucan ≥ 1.45 mg/g, mannan oligosaccharides ≥ 0.33 mg/g, carotenoids ≥ 1.60 mg/g) was provided by the Chinese Academy of Agricultural Sciences Beijing Institute of Animal Husbandry and Veterinary Medicine, which using soybean meal as the solid-state fermentation substrate, and inoculated with the liquid fermentation broth of *Rhodotorula*, the final product contained yeast cell walls, cellular metabolites, and residual medium components. After normal immunization procedures and deworming, the experimental sheep were implemented with single-pen feeding management, fed twice a day, and guaranteed free water.

### 2.2. Sample Collection and Processing

Fecal samples were collected rectally during the final phase of the feeding trial. Some samples were selected using the quarter-sampling approach and stored at −20 °C for subsequent quantitative analysis of the apparent digestibility of nutrients.

After the feeding trial was completed, five sheep were randomly selected from each group for slaughter and sampling. After the sheep were slaughtered, rumen fluid and epithelial tissues were harvested from their rumens. For each individual sheep, 40 mL of rumen content was collected and filtered through four layers of gauze. The resulting rumen fluid was split into two aliquots: one was transferred to a 10 mL cryopreservation tube for subsequent DNA extraction, and the other was placed into a 30 mL centrifuge tube to determine rumen fermentation parameters. Immediately after aliquoting, all samples were transported to the laboratory in liquid nitrogen containers and stored in an ultra-low temperature freezer at −80 °C for subsequent experimental analyses.

### 2.3. Nutrient Apparent Digestibility

The feed and fecal samples were further analyzed for dry matter (DM), CP, EE, Ca, P and acid-insoluble ash (AIA) content. NDF and ADF measurements refer to Wang’s method [32].

The apparent digestibility of nutrients was determined by the endogenous indicator method [33] and 4 mol/L of acid-insoluble ash (4N-AIA) was used as an endogenous indicator for nutrients. The formula is as follows: apparent digestibility of various nutrients (%) = [1 − AB_1_/A_1_B)] × 100%

Note: A is the 4N-AIA content of the diet (%). A_1_ is the 4N hydrochloric acid insoluble ash content of the feces (%). B is the content of a nutrient in the diet (%). B_1_ is the content of a nutrient in the feces (%).

### 2.4. Rumen Histomorphology

Rumen tissue samples were collected from each slaughtered sheep. The samples were trimmed to dimensions of 2 cm by 2 cm and submerged in a 4% paraformaldehyde solution for histological examination. Fixed rumen tissue samples were processed through progressive ethanol dehydration, paraffin embedding, sectioning, and hematoxylin and eosin (H&E) staining. Morphometric measurements, including the length and thickness of the rumen papillae and the thickness of the rumen musculature, were performed using a Nikon Eclipse E200 microscope (Nikon, Tokyo, Japan) and Image View 4 software.

### 2.5. Rumen Fermentation Parameters

In the filtrate of rumen fluid that had undergone preliminary filtration, the pH was measured using a PHS-3C pH meter (INESA, Shanghai, China). NH3-N was fixed with 0.2 mol/L hydrochloric acid, and VFA was fixed with 25% metaphosphoric acid. The remaining rumen fluid was stored in three 15 mL tubes in a refrigerator at −80 °C for future determination of BCP. The determination of BCP was performed using differential centrifugation, ultrasonic disruption to obtain a suspension of rumen microbial protein, staining with Coomassie Blue, and finally, colorimetric determination using an enzyme-linked immunosorbent assay.

### 2.6. High-Throughput Sequencing and Analysis

The 10 mL rumen fluid samples in these cryopreservation tubes were transported via dry ice to Shanghai Meiji Biomedical Co., Ltd. (Shanghai, China) to ensure sample integrity. DNA extraction, PCR amplification and sequencing were performed on 20 samples. Total DNA was extracted from these samples using the Fast DNA™ Spin Kit (MP Biomedicals, Santa Ana, CA, USA). The fungal ITS sequences were amplified by the standard protocol with the primer pair ITS1F/ITS2R (ITS1F: 5′-CTTGGTCATTTAGAGGAAGTAA-3′, ITS2R: 5′-GCTGCGTTCTTCATCGATGC-3′). PCR products were recovered by 2% agarose gel electrophoresis, purified using the AxyPrep DNA Gel Extraction Kit (Axygen Biosciences, Union City, CA, USA), and then eluted with Tris-HCl buffer and analyzed by 2% agarose gel electrophoresis. Detection and quantification were performed with Quanti-Fluor™-ST (Promega, Madison, WI, USA). After quantitative purification, the amplified sequences were sequenced on the Illumina Nextseq 2000 PE300 platform. Raw sequences were processed using QIIME software (version 1.9.1) and clustered into amplicon sequence variants (ASV).

### 2.7. Statistical Analysis

Normality and homoscedasticity of all datasets were assessed via the Shapiro–Wilk’s and Levene’s test, respectively; *p* > 0.05 were deemed to follow a normal distribution [34]. A one-way analysis of variance (ANOVA) was then applied to compare outcomes across the four experimental groups [31]. The model used for the one-way ANOVA can be expressed as

Y ij = μ + τ i + £ ij

Note: where Y ij represents the j-th observed value of the i-th treatment group, μ is the overall mean, τ i is the effect of the i-th treatment group, and £ ij is the random error term.

Subsequent to the one-way ANOVA, Duncan’s multiple range test was utilized for post hoc pairwise comparisons, aiming to pinpoint specific intergroup differences in treatment effects. This test accounts for the multiple comparisons problem through a stepwise comparison of group means, which effectively controls the family-wise error rate throughout the comparative analysis.

All data were presented as mean  ±  standard error. In this study, multiple variables were systematically evaluated, including the apparent digestibility of nutrients, the histomorphology of the rumen, rumen fermentation parameters, and the rumen fungal microbiota. For rumen fungal microbiota data, analyses were performed on the online Majorbio cloud platform (www.majorbio.com, accessed on 24 December 2024). These analyses included alpha diversity analysis, beta diversity analysis and bacterial abundance analysis. The data analysis process involved three main steps: filtering raw data, applying the UPARSE algorithm for clustering, and annotating species. All statistical analyses were performed using IBM SPSS Statistics software (version 26.0; Armonk, NY, USA). All data were presented as mean  ±  standard error. When *p* > 0.05, it indicates that there was no significant difference. When *p* < 0.05, it indicates a significant difference. When *p* < 0.01, it indicates an extremely significant difference.

## 3. Results

### 3.1. Apparent Digestibility of Nutrients

As shown in Table 2, the apparent digestibility of DM, CP, NDF, and ADF was significantly higher in each experimental group compared to CON (*p* < 0.05). The apparent digestibility of CP and ADF was significantly higher in each RYC20-containing experimental group compared to the other groups. (*p* < 0.05). The apparent digestibility of EE, Ca, and P did not differ significantly between the experimental group and the control group.

### 3.2. Rumen Histomorphology

**Figure 1:**
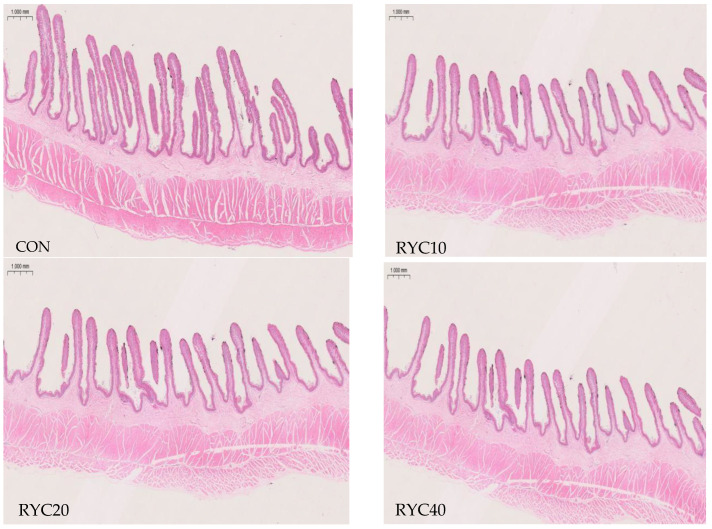
Morphological structure of rumen tissue in sheep, H&E staining of the ruminal wall. CON = fed basal diet; RYC10 = fed basal diet + 10 g/sheep/day RYC; RYC20 = fed basal diet + 20 g/sheep/day RYC; RYC40 = fed basal diet + 40 g/sheep/day RYC. Figure 1 was examined at 100 *×* magnification and a scale bar = 1000 mm.

As shown in Table 3 and Figure 1, compared to the CON group, RYC significantly increased rumen papilla length and muscularis propria thickness in sheep (*p* < 0.05), but had no significant effect on rumen wall thickness (*p* > 0.05). Furthermore, the RYC20 group exhibited significantly greater rumen papilla length and muscularis propria thickness than the other experimental groups (*p* < 0.05).

### 3.3. Rumen Fermentation Parameters

As shown in Table 4, compared with the CON group, the rumen pH and BCP concentrations were significantly higher (*p* < 0.05), while the NH3-N concentration was significantly lower (*p* < 0.05) in the experimental groups. Furthermore, the RYC40 group exhibited significantly higher rumen pH and BCP concentrations, and significantly lower NH3-N concentration, compared to the other experimental groups (*p* < 0.05).

Compared with the CON group, rumen fluid acetate, propionate, butyrate, isobutyric acid, valerate, isovalerate, TVFA and the acetate-to-propionate ratio were significantly higher in the experimental groups than in the CON group (*p* < 0.05). RYC20 rumen acetic acid, propionic acid, butyric acid, isobutyric acid, total volatile fatty acid content and acetate to propionate ratio were significantly higher than the other experimental groups (*p* < 0.05), RYC40 rumen valeric acid content was significantly higher than the other experimental groups (*p* < 0.05), and RYC10 rumen isovaleric acid content was significantly higher than the other experimental groups (*p* < 0.05).

### 3.4. Ruminal Fungal Microbiota

#### 3.4.1. ASV-Based Veen Diagram

**Figure 2:**
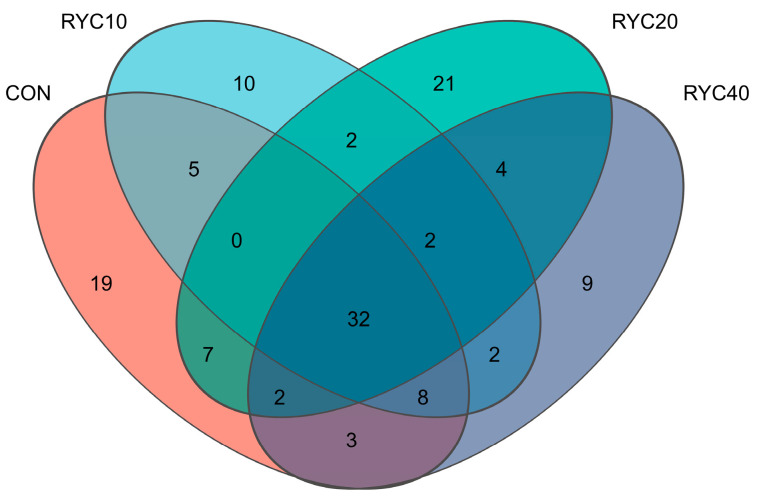
Venn plot of rumen microbiota based on ASV. CON = fed basal diet; RYC10 = fed basal diet + 10 g/sheep/day RYC; RYC20 = fed basal diet + 20 g/sheep/day RYC; RYC40 = fed basal diet + 40 g/sheep/day RYC.

As shown in Figure 2, at the ASV level, the numbers of unique ASVs were 76, 61, 70, and 62 for the CON, RYC10, RYC20, and RYC40 groups, respectively. A core set of 32 ASVs was shared across all groups, indicating a high degree of community similarity and suggesting that dietary RYC supplementation had a limited impact on the rumen fungal community structure.

#### 3.4.2. Alpha Diversity of Rumen Fungal Flora

In the α-diversity analysis, Chao 1 and ACE indices reflect the community richness. Shannon and Simpson indices reflect the community diversity. As shown in Table 5, there was no significant difference in the indices of the experimental groups compared with CON. The coverage rate of each group was higher than 99%, indicating that the sample size was sufficient to reflect the rumen fungal flora of each sheep in different groups. The results indicated that RYC had little impact on rumen fungal diversity in sheep.

#### 3.4.3. β-Diversity of Rumen Fungal Flora

**Figure 3:**
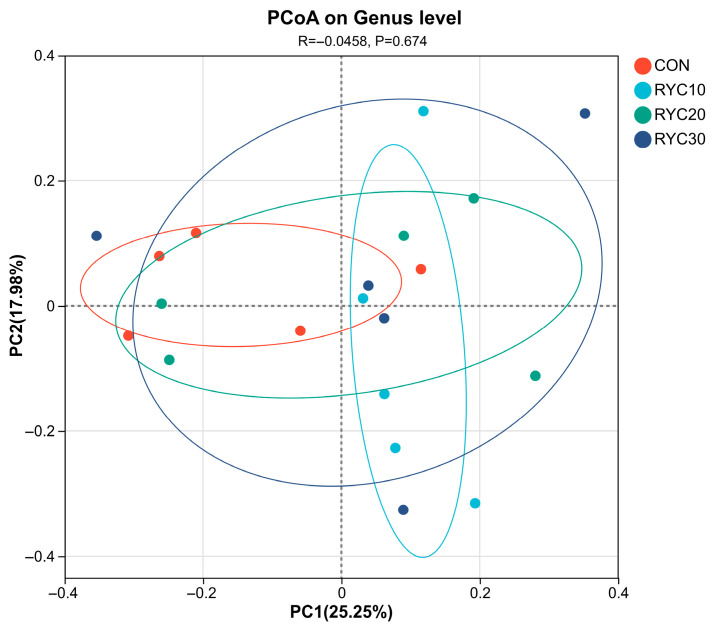
Cluster analysis of rumen microbiota PCoA in sheep. CON = fed basal diet; RYC10 = fed basal diet + 10 g/sheep/day RYC; RYC20 = fed basal diet + 20 g/sheep/day RYC; RYC40 = fed basal diet + 40 g/sheep/day RYC.

As shown in Figure 3, the contribution values of principal component 1 and principal component 2 were 25.25% and 17.98%, respectively. The close proximity of CON to the other groups in this experiment suggests that the difference between it and the rumen micro-ruminal fungal flora of the experimental group is not obvious.

**Figure 4:**
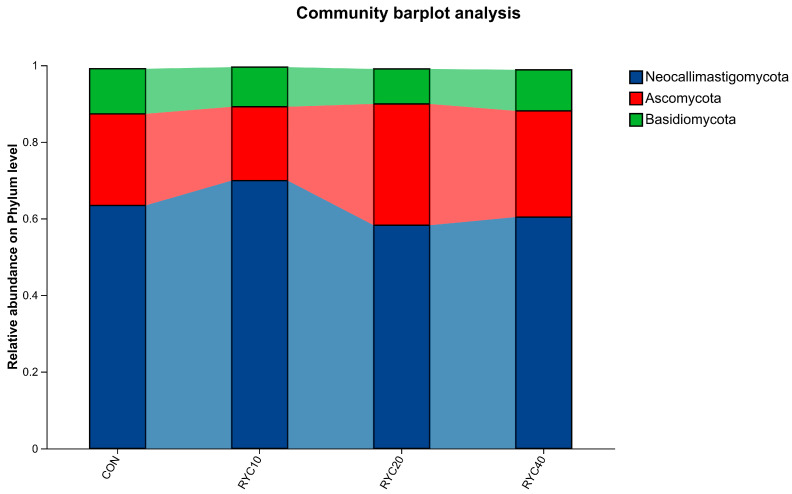
Level composition of rumen microbiota in sheep. CON = fed basal diet; RYC10 = fed basal diet + 10 g/sheep/day RYC; RYC20 = fed basal diet + 20 g/sheep/day RYC; RYC40 = fed basal diet + 40 g/sheep/day RYC. Only the categories that are representative in the graph should be included.

As shown in Figure 4 and Table 6, at the phylum level, the rumen fungal communities in both the CON and RYC-supplemented groups were predominantly composed of *Neocallimastigomycota*, *Basidiomycota*, and *Ascomycota*. Their relative abundances in the CON group were 63.40%, 23.93%, and 11.77%, respectively; corresponding values were 69.89%, 19.27%, and 10.35% in RYC10; 58.24%, 31.67%, and 9.14% in RYC20; and 60.36%, 27.73%, and 10.74% in RYC40. Statistical analysis revealed that RYC supplementation significantly increased the abundances of *Neocallimastigomycota* and *Ascomycota* (*p* < 0.05), while no significant differences were observed among the remaining phyla.

**Figure 5:**
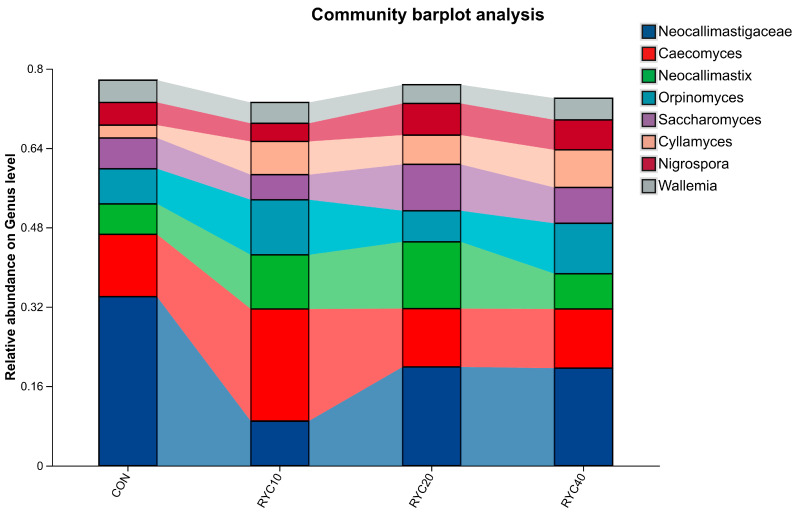
Horizontal composition of rumen microbiota in sheep. CON = fed basal diet; RYC10 = fed basal diet + 10 g/sheep/day RYC; RYC20 = fed basal diet + 20 g/sheep/day RYC; RYC40 = fed basal diet + 40 g/sheep/day RYC. Only the categories that are representative in the graph should be included.

As shown in Figure 5 and Table 7, differential analysis at the genus level indicated that RYC supplementation induced variations in the fungal community composition of rumen fluid. The genera of fungi that were co-dominant in CON and the experimental groups were *Neocallimastigaceae*, *Caecomyces*, *Neocallimastix* and *Orpinomyces*. Among them, the proportion of CON was 34.02%, 12.57%, 6.16%, 7.08%; RYC10 was 8.95%, 22.61%, 10.90%, 11.12%; RYC20 was 19.86%, 11.76%, 13.46%, 6.28%. RYC40 was 19.61%, 11.96%, 7.09%, 10.12%. Dietary supplementation with RYC significantly increased the relative abundance of the fungal genera *Neocallimastix*, *Orpinomyces, Nigrospora*, *Saccharomyces*, and *Cyllamyces* in the rumen (*p* < 0.05).

### 3.5. Dose–Response Patterns of RYC Supplementation on Nutrient Digestibility, Rumen Function and Rumen Fungal Microbiota

We systematically summarized and analyzed the dose–response trends of all major measured parameters (including nutrient apparent digestibility, rumen histomorphology, rumen fermentation indices, and rumen microbiota composition) across the RYC supplementation gradients (0, 10, 20, 40 g/d).

Specifically, we clarified the divergent response trends of different parameter categories: ① nutrient digestibility (DM, CP, NDF, ADF) and rumen histomorphology (papillary length, muscular layer thickness) exhibited a peaked dose–response pattern, with optimal performance at 20 g/d and a slight decline at 40 g/d; ② partial rumen fermentation indices showed a mild increasing trend with increasing RYC dosage (with 40 g/d yielding relatively higher values for individual fermentation traits); ③ rumen microbiota (especially the relative abundance of core fibrolytic fungi) responded positively to RYC supplementation within the range of 0–20 g/d and remained stable at 40 g/d without a further significant increase.

## 4. Discussion

### 4.1. Apparent Digestibility of Nutrients

Apparent nutrient digestibility is a critical indicator for evaluating feed nutritional value and animal digestive function [35], as it directly influences growth performance. The apparent digestibility of nutrients is used to evaluate the efficiency of nutrient absorption and utilization by animals from diets. The level of apparent digestibility is directly related to animal production performance, such as growth rate, laying rate, milk yield and so on. Nutrient digestibility is one of the essential indicators in animal production, which holds great practical significance and provides important reference for livestock producers to make rational decisions. Its measurement and analysis have important guiding significance for reducing feed waste, improving production efficiency and making economic decisions for livestock producers. Studies have demonstrated that YC can enhance this parameter; as a natural product from yeast fermentation, YC has been shown in ruminant studies to enhance feed digestibility, feed intake and growth performance in such animals [36,37]. For instance, Wohlt et al. [38] reported increased dry matter intake, milk yield, and apparent digestibility of CP and fiber in dairy cows supplemented with YC. Similarly, Malekkhahi et al. [39] observed significant improvements in the apparent digestibility of CP and neutral detergent fiber (NDF) when YC was added to highly concentrated diets. Furthermore, YC supplementation has been shown to boost dietary fiber digestibility [40]. These improvements are often attributed to YC’s ability to stimulate rumen cellulolytic bacteria by promoting a more active degradative microflora and metabolite production [41,42], as well as by enhancing the colonization of anaerobic fungi on fibrous substrates, which increases their accessibility to fibrolytic bacteria [43].

In this study, all RYC-supplemented groups exhibited significantly increased apparent digestibility of CP, NDF and ADF, with maximal improvement observed at 20 g/d. The crude fiber degradation capacity was evaluated by determining the digestibility of ADF and NDF, which are key parameters reflecting fiber digestion status. NDF includes total fiber components such as hemicellulose, cellulose, and lignin, while ADF mainly represents recalcitrant fractions such as cellulose and lignin. Therefore, the simultaneous improvement in the digestibility of both indicators can well reflect the promoting effect of RYC on crude fiber degradation. Specifically, our data showed that the ADF digestibility in the RYC20 group was significantly higher than that in the CON group. Furthermore, the results of fungal community analysis further supported the fiber degradation function of RYC: the abundance of the phylum *Neocallimastigomycota* was significantly increased, and potent fiber-degrading fungal genera such as *Cyllamyces* and *Neocallimastix* also showed a significant enrichment trend. However, this promoting effect exhibited an obvious dose threshold. The decline in the abundances of the aforementioned key genera in the RYC40 group was consistent with the reduction in nutrient digestibility, suggesting that excessive supplementation may be counterproductive. These findings are consistent with the aforementioned YC-related research results. While this dose-dependent response aligns with established YC effects, the distinct *Rhodotorula*-specific mechanisms warrant further investigation.

### 4.2. Rumen Histomorphology

Diet composition, concentrate/crude ratio and the physical form of the diet are the main factors affecting the development of rumen papillae [44,45]. Steele [46] demonstrated that high-concentrate diets resulted in a significantly lower rumen wall thickness in beef cattle than the thickness resulting from the low-concentrate group. Similarly, Lesmeister et al. [47] established an evaluation system based on a systematic analysis and pointed out that the length and width of the papillae and the thickness of the rumen wall were the key morphological indicators reflecting the degree of rumen development in young ruminants. These parameters are significantly and positively correlated with nutrient absorption efficiency and fermentation function.

Ruminal development in ruminants is driven by both physical and chemical regulatory mechanisms: the former through mechanical friction of roughage to stimulate myofibrillar proliferation and the latter relying on microbial metabolites, such as volatile fatty acids, to stimulate ruminal epithelial development [48].

In this study, dietary addition of RYC resulted in highly significant increases in both rumen papilla length and muscularis layer thickness in sheep. These results suggest that RYC may promote rumen development and papilla surface area in lambs by directly or indirectly stimulating rumen epithelial cell proliferation and differentiation. This enhancement is likely facilitated by improved feed utilization, which supplies essential substrates for rumen tissue growth [49].

### 4.3. Rumen Fermentation Parameters

The rumen, as the primary digestive organ of ruminants, hosts complex microbial communities that synergistically provide essential nutrients [50]. While high-concentrate diets enhance energy availability, they predispose ruminants to lactic acid accumulation and pH depression, triggering subacute ruminal acidosis (SARA) [51]. Ruminal NH_3_-N serves as the primary nitrogen source for microbial protein synthesis [52]. YC counters these effects through multiple mechanisms: its organic acids, B vitamins and functional polysaccharides, which selectively stimulate cellulolytic bacteria proliferation [53], thereby modulating ruminal microbiota structure. Concurrently, YC stabilizes ruminal pH within the optimal 6.2–6.8 range while increasing total volatile fatty acid (TVFA) production by 15–22% [54].

In this study, our results corroborate these effects, demonstrating significantly elevated ruminal pH (*p* < 0.01) in RYC-supplemented groups versus controls (CON). The 40 g/d RYC dose optimally regulated ammonia-nitrogen (NH_3_-N) concentrations, consistent with Erasmus et al. [55], suggesting enhanced bacterial crude protein (BCP) synthesis via optimized nitrogen metabolism. Furthermore, RYC supplementation altered fermentation patterns, reducing the ratio of acetate and propionate through increased propionate production—aligning with Chaucheyras et al.’s observations on yeast-mediated rumen modulation [53]. It should be noted that the total volatile fatty acid (TVFA) concentrations measured in the present study were relatively low, which might be associated with the following experimental conditions: (1) sampling time point: rumen fluid was collected before morning feeding (in a fasted state), at which time VFA concentrations are typically at the diurnal low level; (2) diet type: a diet with a high forage proportion (Wildrye + corn stover accounting for 42%) was adopted in this study. Although such a high-forage diet results in relatively low VFA production, it can maintain a more stable fermentation status; (3) animal status: the rumen development and fermentation function of three-month-old lambs are not yet fully mature, leading to a lower VFA production capacity compared with adult ruminants. Nevertheless, RYC supplementation still significantly increased the TVFA content (*p* < 0.05), and the VFA proportion pattern in each treatment group was consistent with the characteristics of high-forage diets (a high acetate/propionate ratio), indicating a normal rumen fermentation function.

This dose-dependent response may reflect *Rhodotorula* strain specificity: its unique metabolites potentially favor acetate-producing microbiota, whereas conventional yeasts preferentially stimulate propionate pathways. Notably, elevated branched-chain VFAs (isobutyrate, valerate) indicate activated proteolytic flora [56], offering novel strategies for amino acid balance optimization in high-concentrate systems. Nevertheless, strain-specific metabolic mechanisms require further validation through metagenomic and multi-omic approaches.

### 4.4. Ruminal Fungal Microbiota

The rumen is colonized by bacteria, protozoa, archaea, fungi and viruses that degrade complex plant fibers and polysaccharides and produce VFA, microbial proteins and vitamins, which in turn provide nutrients to meet host maintenance and growth requirements [57]. The rumen fungal community composition of ruminants is a dynamic process influenced by multiple factors, including diet and age [14,58,59]. Anaerobic fungi occupy a unique ecological niche in the gastrointestinal tract of large herbivores and are recognized as the major force responsible for plant cell wall degradation during the digestive process. They secrete high levels of cellulolytic and hemicellulolytic enzymes that penetrate the cuticle to decompose plant cell walls, thereby facilitating the breakdown and utilization of fiber by bacteria [60,61]. In adult ruminants, rumen fungi account for 3% to 4% of the total rumen microbial biomass, and the absence of these fungi leads to a significant reduction in the degradation rate of rumen cellulose [62]. Compared to studies on rumen bacteria and archaea, there have been few studies on rumen fungi [63]. In recent years, researchers have begun to pay attention to the study of rumen fungi, and several genera of fungi have been isolated from the rumen, including *Neocallimastix*, *Caecomyces*, *Piromyces*, *Orpinomyces*, *Anaeromyces*, and *Cyllamyces* [64].

Analysis of the Alpha and Beta diversity of the ruminal fungal flora revealed that dietary RYC supplementation had no significant effect on its diversity. RYC selectively enhances the growth of core fibrolytic fungi in the rumen without altering the overall diversity and community structure of the rumen microbiota. In this study, the rumen fungal community in sheep was predominantly composed of the phyla *Neocallimastigomycota* and *Ascomycota*. *Neocallimastigomycota* comprises anaerobic fungi that inhabit the rumen and digestive tract of herbivores. These fungi were capable of enzymatically degrading lignin, cellulose, and hemicellulose substrates [65]. When ruminants consumed high-fiber diets, these fungi secreted a range of fibrolytic enzymes that effectively broke down non-lignified plant cell walls [66]. Owing to their strong penetrating ability, they invaded fibrous plant tissues, reduced internal tension, and loosened the structure, thereby facilitating subsequent degradation by other microorganisms—a process in which they played a synergistic role [67,68]. *Ascomycota*, the most diverse fungal phylum, was primarily responsible for the decomposition of recalcitrant organic compounds such as lignin and keratin [69]. The increased abundance of these fungal genera suggested that RYC supplementation likely enhanced the digestion of dietary fiber in the rumen. RYC resulted in an upward trend in the abundance of *Neocallimastigomycota* and *Ascomycota* in the rumen. It can be hypothesized that RYC promotes the growth of *Neocallimastigomycota* and *Ascomycota* microorganisms and accelerates the degradation of fibrous substances in the diet. It can also improve the digestibility of nutrients in animals.

At the genus level, the ruminal fungal community in sheep was primarily composed of *Neocallimastigaceae*, *Caecomyces*, *Neocallimastix*, and *Orpinomyces*, which is consistent with the findings reported by Kittelmann et al. [70]. Among these, *Neocallimastix* and *Piromyces* exhibited stronger straw-degrading capabilities, whereas *Caecomyces* showed relatively weaker fibrolytic activity. Dietary supplementation with RYC significantly increased the abundance of *Neocallimastix*, *Orpinomyces*, *Nigrospora*, *Saccharomyces*, and *Cyllamyces* in the rumen. The strong penetrating ability of *Neocallimastix* contributed to the disruption of plant fiber structure, facilitating synergistic degradation by other microorganisms. *Orpinomyces*, an anaerobic fungal genus within the *Neocallimastigomycota*, commonly resides in the digestive tract of herbivorous livestock and plays a significant role in decomposing plant residues [71]. *Nigrospora* was also identified as capable of promoting dietary fiber decomposition. Additionally, *Cyllamyces*, another gastrointestinal anaerobic fungus, contributes markedly to fiber digestion through the production of enzymes that break down cellulose, xylan, and starch [72]. *Rhodotorula* sp. (the strain of RYC) synthesizes and accumulates a variety of bioactive substances during growth, including carotenoids, extracellular polysaccharides, organic acids and B vitamins. Among them, extracellular polysaccharides and organic acids can serve as specific, easily utilizable carbon and energy substrates for rumen anaerobic fibrolytic fungi, which are difficult to decompose with complex structural carbohydrates alone in the early growth stage; B vitamins act as essential growth factors for the proliferation and metabolic activity of anaerobic fungi (which lack the ability to synthesize most B vitamins de novo). RYC thus provides direct nutritional support for the growth of fibrolytic fungi, promoting their early colonization and proliferation in the rumen microenvironment. The carotenoids and extracellular metabolites of RYC exert a prebiotic effect that selectively modulates the rumen microbial niche. Specifically, the potent antioxidant activity of RYC carotenoids alleviates oxidative stress in rumen fibrolytic fungi. Anaerobic fungi are highly sensitive to reactive oxygen species (ROS) in the rumen, and oxidative stress can significantly inhibit the expression of their fiber-degrading enzyme genes (e.g., cellulase, xylanase) and hyphal growth [31]. Meanwhile, RYC metabolites do not provide a suitable nutritional niche for non-fibrolytic microbes (e.g., some opportunistic bacteria), thus avoiding nutrient competition and achieving the selective enrichment of fibrolytic fungi.

Analysis of the experimental data indicated that RYC supplementation at 20 g/d notably altered the fungal community. Specifically, it robustly increased the relative abundance of *Cyllamyces* and significantly increased that of *Neocallimastix*—two genera renowned for their role in degrading fibrous material. This aligns with established research demonstrating the capacity of YC metabolites to promote fibrolytic bacterial communities [73].

Our results are consistent with the findings of Fliegerova et al. [4] that anaerobic fibrolytic fungi are the core functional microbes for rumen cellulose/hemicellulose degradation, and their abundance is positively correlated with the rumen fiber digestibility, Fliegerova et al. reported that high-concentrate diets inhibit the growth of anaerobic fungi and reduce fiber degradation efficiency in goat rumen; our study further complements this finding by demonstrating that RYC supplementation can selectively enrich the core anaerobic fibrolytic fungi *(Neocallimastix, Orpinomyces, Nigrospora, Saccharomyces, and Cyllamyces*) and improve rumen fiber degradation capacity, providing a feasible feed additive strategy for the regulation of rumen fibrolytic fungal microbiota in intensive ruminant production (where high-concentrate diets are commonly used). Combined with previous data on the apparent digestibility of nutrients, the presence of these fungi significantly improved the utilization rate of fibrous substances in the diet by sheep. As discussed in the section on apparent digestibility of nutrients, the mechanisms of RYC and YC are similar. RYC-induced facilitation of anaerobic fungal colonization on fibrous substrates enhances accessibility for fibrolytic bacteria. In future research, in-depth investigations (e.g., transcriptomic analysis of fiber-degrading fungi) will be required to further validate the underlying mechanisms.

### 4.5. Dose–Response Patterns of RYC Supplementation on Nutrient Digestibility, Rumen Function and Rumen Fungal Microbiota

We first clarify that nutrient apparent digestibility and rumen histomorphology are the core biological parameters for evaluating the practical application effect of feed additives in ruminant production, as they directly determine the nutrient utilization efficiency and long-term rumen health of animals—two key factors that affect the overall production performance and economic benefits of ruminants. Although partial fermentation indices showed relatively higher values at 40 g/d, these single fermentation traits did not translate into improved nutrient digestibility or rumen development, and their biological significance for the overall production performance is secondary compared with digestibility and rumen morphology.

RYC supplementation at 40 g/d led to a slight increase in individual rumen fermentation indices but caused a significant decline in nutrient digestibility and led to no further improvement in rumen histomorphology and core fibrolytic fungi abundance, which is attributed to the mild nutrient competition and rumen micro-ecological imbalance caused by excessive RYC addition (as elaborated in the previous response). In contrast, 20 g/d of RYC achieved the optimal performance in the core biological parameters (digestibility and rumen morphology) while maintaining favorable fermentation characteristics and microbial community structure, with no obvious negative trade-offs between all measured parameters.

Based on the actual market price of RYC and the measured improvement in nutrient digestibility at 20 g/d, we calculate that the increase in feed cost caused by doubling RYC dosage from 10 g/d to 20 g/d can be fully offset by the economic benefits brought by the significant improvement in nutrient utilization efficiency and rumen health (we have supplemented the specific cost–benefit calculation basis and data in the revised manuscript). In addition, 40 g/d of RYC not only fails to further improve the core production performance but also significantly increases the feed cost without additional economic returns, which is not feasible for large-scale practical application 20 g/d RYC.

## 5. Conclusions

Supplementation of RYC at 20 g/d optimizes apparent nutrient digestibility and rumen tissue development in ruminants while maintaining favorable rumen fermentation characteristics and selectively enhancing the growth of core fibrolytic fungi; this dosage achieves the optimal balance of biological performance and economic feasibility and is thus recommended as the optimal practical supplementation dosage for ruminant production.

## Figures and Tables

**Table 1:** Basic diet composition and nutrient levels (dry matter basis).

Items	Content (%)
Ingredients	
Sheep grass	9.21
Corn talks	32.90
Concentrate supplements	31.57
Whole plant corn silage	26.32
Total	100.00
Nutrients level	
Metabolic energy (MJ/kg)	8.09
Crude protein (CP)	11.95
Ether extract (EE)	2.13
Neutral detergent fibers (NDF)	54.86
Acidic detergent fibers (ADF)	21.60
Calcium (Ca)	0.69
Phosphorus (P)	0.25

Note: Metabolizable energy is calculated; the rest of the nutrients are measured.

**Table 2:** Effect of RYC on apparent digestibility of nutrients of sheep.

Items	CON	RYC10	RYC20	RYC40	*p*-Value
DM	65.22 ± 4.47 ^c^	68.08 ± 4.15 ^b^	70.36 ± 5.02 ^a^	69.87 ± 4.98 ^ab^	0.046
CP	60.27 ± 0.29 ^d^	63.24 ± 0.26 ^c^	66.58 ± 0.43 ^a^	64.83 ± 0.46 ^b^	<0.01
EE	74.05 ± 0.44	76.40 ± 0.59	82.18 ± 1.01	80.21 ± 0.26	0.083
NDF	51.84 ± 0.28 ^c^	57.24 ± 0.73 ^b^	62.84 ± 0.38 ^a^	60.46 ± 4.91 ^ab^	<0.01
ADF	51.31 ± 0.49 ^c^	62.78 ± 0.80 ^b^	73.56 ± 0.52 ^a^	63.28 ± 0.96 ^b^	<0.01
Ca	42.04 ± 0.76	42.31 ± 0.58	42.52 ± 0.51	42.66 ± 0.47	0.61
P	55.63 ± 0.12	56.17 ± 0.18	56.39 ± 0.19	56.28 ± 0.40	0.073

Note: data represent mean ± standard error (SE) (*n* = 5). Different superscript letters (^a, b, c, d^) within a row denote significant differences (*p* < 0.05) according to Duncan’s multiple range test, CON = fed basal diet; RYC10 = fed basal diet + 10 g/sheep/day RYC; RYC20 = fed basal diet + 20 g/sheep/day RYC; RYC40 = fed basal diet + 40 g/sheep/day RYC.

**Table 3:** Effect of RYC on rumen morphology in sheep.

Items	CON	RYC10	RYC20	RYC40	*p*-Value
Ruminal papilla length (mm)	2.26 ± 0.02 ^c^	2.35 ± 0.02 ^b^	2.66 ± 0.01 ^a^	2.46 ± 0.01 ^b^	0.024
Rumen wall thickness (mm)	2.27 ± 0.01	2.30 ± 0.01	2.45 ± 0.04	2.29 ± 0.04	0.064
Muscle layer thickness (mm)	0.57 ± 0.01 ^d^	0.63 ± 0.01 ^b^	0.67 ± 0.01 ^a^	0.60 ± 0.01 ^c^	0.018

Note: data represent mean ± standard error (SE) (*n* = 5). Different superscript letters (^a, b, c, d^) within a row denote significant differences (*p* < 0.05) according to Duncan’s multiple range test, CON = fed basal diet; RYC10 = fed basal diet + 10 g/sheep/day RYC; RYC20 = fed basal diet + 20 g/sheep/day RYC; RYC40 = fed basal diet + 40 g/sheep/day RYC.

**Table 4:** Effects of RYC on rumen fermentation in sheep.

Items	CON	RYC10	RYC20	RYC40	*p*-Value
PH	6.49 ± 0.01 ^d^	6.68 ± 0.02 ^c^	6.94 ± 0.02 ^b^	7.10 ± 0.01 ^a^	<0.01
NH_3_-N (mg/dL)	17.18 ± 0.48 ^a^	16.18 ± 0.34 ^b^	15.36 ± 0.44 ^c^	14.29 ± 0.55 ^d^	<0.01
BCP (mg/dL)	1.47 ± 0.02 ^d^	1.52 ± 0.01 ^c^	1.58 ± 0.01 ^b^	1.7 ± 0.01 ^a^	<0.01
Acetate (mmol/L)	28.93 ± 0.66 ^d^	37.42 ± 0.54 ^b^	43.53 ± 0.71 ^a^	32.38 ± 0.7 ^c^	<0.01
Propionate (mmol/L)	6.23 ± 0.40 ^d^	7.40 ± 0.12 ^b^	8.24 ± 0.03 ^a^	6.61 ± 0.17 ^c^	<0.01
Butyrate (mmol/L)	3.79 ± 0.08 ^d^	4.55 ± 0.08 ^c^	6.21 ± 0.05 ^a^	5.49 ± 0.15 ^b^	<0.01
Isobutyric acid (mmol/L)	0.39 ± 0.01 ^d^	0.43 ± 0.04 ^c^	0.60 ± 0.03 ^a^	0.53 ± 0.01 ^b^	<0.01
Valerate (mmol/L)	0.29 ± 0.02 ^d^	0.32 ± 0.02 ^c^	0.35 ± 0.02 ^b^	0.41 ± 0.01 ^a^	<0.01
Isovalerate (mmol/L)	0.40 ± 0.01 ^d^	0.57 ± 0.01 ^a^	0.50 ± 0.12 ^b^	0.46 ± 0.01 ^c^	<0.01
Acetate/Propionate	4.64 ± 0.14 ^d^	5.05 ± 0.02 ^b^	5.28 ± 0.09 ^a^	5.48 ± 0.05 ^c^	<0.01
TVFA (mmol/L)	39.23 ± 0.76 ^c^	50.68 ± 0.78 ^ab^	59.25 ± 0.77 ^a^	45.43 ± 0.82 ^b^	<0.01

Note: data represent mean ± standard error (SE) (*n* = 5). Different superscript letters (^a, b, c, d^) within a row denote significant differences (*p* < 0.05) according to Duncan’s multiple range test, CON = fed basal diet; RYC10 = fed basal diet + 10 g/sheep/day RYC; RYC20 = fed basal diet + 20 g/sheep/day RYC; RYC40 = fed basal diet + 40 g/sheep/day RYC.

**Table 5:** Alpha analysis of rumen in sheep.

Items	CON	RYC10	RYC20	RYC40	*p*-Value
ACE	56.51 ± 5.81	56.54 ± 8.28	66.47 ± 10.86	58.2 ± 6.21	0.627
Chao 1	17.8 ± 2.71	21.2 ± 2.23	24.40 ± 5.88	22.20 ± 4.92	0.818
Shannon	2.77 ± 0.28	2.94 ± 0.38	3.00 ± 0.35	2.96 ± 0.48	0.634
Simpson	0.13 ± 0.05	0.12 ± 0.08	0.09 ± 0.05	0.11 ± 0.06	0.506
Coverage	99.12	99.56	99.79	99.99	0.049

Note: data represent mean ± standard error (SE) (*n* = 5). CON = fed basal diet; RYC10 = fed basal diet + 10 g/sheep/day RYC; RYC20 = fed basal diet + 20 g/sheep/day RYC; RYC40 = fed basal diet + 40 g/sheep/day RYC.

**Table 6:** Effects of adding RYC to the diet on the level of rumen fungal phylum in sheep.

Items	CON	RYC10	RYC20	RYC40	*p*-Value
*Neocallimastigomycota*	63.40 ^c^	69.89 ^b^	75.24 ^a^	65.36 ^b^	0.027
*Ascomycota*	21.93 ^b^	22.27 ^a^	24.67 ^a^	22.73 ^a^	0.012
*Basidiomycot*	11.77	10.35	9.14	10.74	0.335

Note: data represent mean ± standard error (SE) (*n* = 5). Different superscript letters (^a, b, c^) within a row denote significant differences (*p* < 0.05) according to Duncan’s multiple range test, CON = fed basal diet; RYC10 = fed basal diet + 10 g/sheep/day RYC; RYC20 = fed basal diet + 20 g/sheep/day RYC; RYC40 = fed basal diet + 40 g/sheep/day RYC.

**Table 7:** Effects of adding RYC to the diet on the genus level of rumen fungal communities in sheep.

Items	CON	RYC10	RYC20	RYC40	*p*-Value
*Neocallimastigaceae*	34.02	8.95	19.86	19.61	0.236
*Caecomyces*	12.57	22.61	11.76	11.96	0.612
*Neocallimastix*	6.16 ^c^	10.90 ^ab^	13.46 ^a^	7.09 ^b^	0.018
*Orpinomyces*	7.08 ^c^	11.12 ^a^	9.28 b	10.16 ^b^	0.031
*Saccharomyces*	1.73 ^c^	5.05 ^b^	6.35 ^ab^	7.22 ^a^	0.029
*Cyllamyces*	2.58 ^d^	4.69 ^c^	7.9 ^a^	7.60 ^b^	0.003
*Nigrospora*	4.59 ^c^	5.67 ^b^	6.38 ^a^	6.05 ^ab^	0.047
*Wallemia*	4.49	4.20	3.77	4.38	0.354

Note: data represent mean ± standard error (SE) (*n* = 5). Different superscript letters (^a, b, c, d^) within a row denote significant differences (*p* < 0.05) according to Duncan’s multiple range test, CON = fed basal diet; RYC10 = fed basal diet + 10 g/sheep/day RYC; RYC20 = fed basal diet + 20 g/sheep/day RYC; RYC40 = fed basal diet + 40 g/sheep/day RYC.

## Data Availability

The original contributions presented in this study are included in the article material.
